# Revisiting low penetrance retinoblastoma: an integrated clinical, genetic, and bioinformatic analysis

**DOI:** 10.1093/hmg/ddag026

**Published:** 2026-04-04

**Authors:** Eden Avnat, Guy Shapira, Yael Lustig, Jonathan Citrin, Duangnate Rojanaporn, Rossukon Kaewkhaw, Dong Hyun Jo, Jeong Hun Kim, Noam Shomron, Eitan Friedman, Ido Didi Fabian

**Affiliations:** Gray Faculty of Medicine, Tel Aviv University, Tel Aviv 69978, Israel; Gray Faculty of Medicine, Tel Aviv University, Tel Aviv 69978, Israel; Edmond J. Safra Center for Bioinformatics, Tel Aviv University, Tel Aviv 69978, Israel; The Goldschleger Eye Institute, Sheba Medical Center, Tel Hashomer, Ramat Gan 62521, Israel; Faculty of Medicine, Hebrew University of Jerusalem, Jerusalem 9112102, Israel; Department of Ophthalmology, Faculty of Medicine, Ramathibodi Hospital, Mahidol University, 270 Rama VI Road, Thung Phaya Thai, Ratchathewi, Bangkok 10400, Thailand; Chakri Naruebodindra Medical Institute, Faculty of Medicine Ramathibodi Hospital, 111 Moo 14, Tambon Bang Pla, Amphoe Bang Phli, Samut Prakan 10540, Thailand; Program in Translational Medicine, Faculty of Medicine Ramathibodi Hospital, Mahidol University, 270 Rama VI Road, Thung Phaya Thai, Ratchathewi, Bangkok 10400, Thailand; Department of Anatomy and Cell Biology, Seoul National University College of Medicine, 103 Daehak-ro, Jongno-gu, Seoul 03080, Republic of Korea; Department of Biomedical Sciences and Ophthalmology, Seoul National University College of Medicine, 103 Daehak-ro, Jongno-gu, Seoul 03080, Republic of Korea; Global Excellence Center for Gene and Cell Therapy, Seoul Narional University Hospital, 103 Daehak-ro, Jongno-gu, Seoul 03080, Republic of Korea; Gray Faculty of Medicine, Tel Aviv University, Tel Aviv 69978, Israel; Edmond J. Safra Center for Bioinformatics, Tel Aviv University, Tel Aviv 69978, Israel; Gray Faculty of Medicine, Tel Aviv University, Tel Aviv 69978, Israel; The Meirav High Risk Clinic, Chaim Sheba Medical Center, Tel-Hashomer, Ramat Gan 52621, Israel; The Goldschleger Eye Institute, Sheba Medical Center, Tel Hashomer, Ramat Gan 62521, Israel; International Centre for Eye Health, London School of Hygiene & Tropical Medicine, Keppel Street, London, WC1E 7HT, United Kingdom

**Keywords:** Retinoblastoma, Low-Penetrance, Hypomorphic variants, Pathogenic sequence variants—PSV

## Abstract

Retinoblastoma (RB) is typically associated with highly penetrant pathogenic sequence variants (PSVs) in the *RB1* gene; however, some families exhibit low penetrance RB (LPRB). We aimed to determine the penetrance rate and identify genetic and clinical characteristics of LPRB. To that end two cohorts were analyzed: 250 genetically confirmed LPRB cases identified through systematic literature review and 78 classical germline RB (CGRB) from three international centers- Thailand, Korea, and Israel. Penetrance rate was estimated as the proportion of affected individuals among *RB1* PSV carriers. Multivariate models assessed parent-of-origin effects and predictors of penetrance. PSVs were annotated with Combined Annotation Dependent Depletion (CADD) scores and mapped to pRB structural domains. LPRB penetrance ranged from 50% (125/250, non-age-adjusted, CI [43.8%–56.2%]) to 64% (125/196, age-adjusted, CI [56.8%–70.2%]). Paternal inheritance of *RB1* PSV was associated with a significantly increased risk of LPRB in offspring (OR = 6.24; *P* < 0.0001). Clinically, LPRB were significantly more likely than CGRB to present with unilateral disease (OR = 9.3, *P* < 0.0001), diagnosed at an older age (13 Vs 6.5 months, *P* = 0.01), and affect males (OR = 2.4, *P* = 0.03). LPRB-associated PSVs showed lower CADD scores (OR = 1.5; *P* = 0.0008), indicating lower predicted pathogenicity, and were enriched in pRB’s N- or C-terminal domains (OR = 3.2; *P* = 0.007), consistent with hypomorphic effects. In conclusion, LPRB shows a 50–64% penetrance rate, more likely to be paternally inherited, have unilateral presentation, and associated with hypomorphic *RB1* PSVs in the terminal pRB regions. These findings support retitling ‘low penetrance RB’ to ‘medium penetrance RB’.

## Introduction

Retinoblastoma (RB) [MIM:180200], the most frequent childhood ocular cancer, is associated in ~ 40% of cases with germline pathogenic sequence variants (PSVs) in the *RB1* gene [1]. This autosomal dominant inherited cancer syndrome is considered a model for high penetrant mutant cancer susceptibility alleles, estimated at > 90% [1, 2]. Yet, in some families, penetrance is lower, with mutant alleles referred to as low penetrance *RB1* (LPRB) alleles [2, 3].

Several mechanisms that may explain the origin of these LPRB alleles have been proposed and studied: effect of PSV location on protein binding activity, while retaining other RB protein functions and functional domains [4, 5], loss of E2F binding capability and hypo-phosphorylation [6], unstable, temperature-sensitive pocket protein-binding activity [7], use of an alternative initiation site generating a shorter yet partially active protein [8], parent of origin effect via methylation [9, 10], modifier genes [9] and somatic mosaicism [10].

Thus, the prevailing paradigm suggests that LPRB alleles arise from PSVs that either reduce the quantity of normal RB protein produced (class 1 PSVs) or result in a partially functional mutant RB protein (class 2 PSVs) [2, 3, 5, 6, 9]. However, only few functional assays have directly validated these models, and no systematic comparison of clinical, genetic, and pRB domain-focused differences between LPRB and classical (high-penetrance) germline RB (CGRB) has yet been published.

In this study, the primary objective was to estimate the penetrance rate of LPRB. Additionally, we aimed to better characterize LPRB by comparing the clinical, genetic, and molecular features of LPRB PSVs to a cohort of CGRB patients.

## Results

### LPRB cohort and penetrance rate

**Figure 1:**
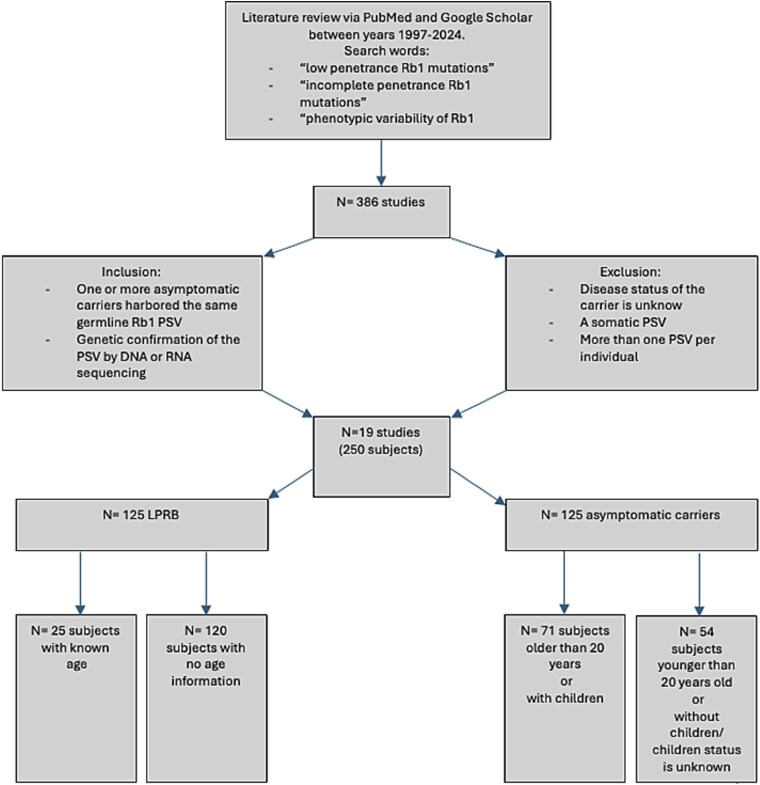
CONSORT-style flow diagram of the LPRB cohort. The flow chart describes the steps taken in the current study to identify previously published LPRB cases and the selection process for inclusion in subsequent analysis.

Based on our systematic literature review, 19 of 386 screened studies met all eligibility inclusion criteria and were used to construct the LPRB cohort (Fig. 1). This cohort comprised 250 individuals with a germline *RB1* PSV, of whom 58% (118/204) were males, 65% (79/121) had unilateral disease, the median age at time of diagnosis was 13 months [6–24] and the median raw CADD score was 5.7 [4.8–6.2] (Table 1).

**Table 1:** Low penetrance retinoblastoma and classical germline retinoblastoma cohorts.

		Low penetrance Retinoblastoma N = 250	Classical Germline Retinoblastoma N = 78		Total N = 328
Sex	Female	42%, (86/204^a^)	53% (41/78)	*P* = 0.15	45%, (127/282)
	Male	58%, (118/204^a^)	47% (37/78)	*P* = 0.15	55%, (155/282)
	Missing data	18%, (46/250)	0% (0/78)	-	14%, (46/328)
Age at diagnosis (in month)		13 [6–24] (24/24^a^)	6.5 [3–14] (78/78)	*P* = 0.01	8 [3–18.5]
Laterality	Unilateral	65%, (79/121^a^)	15%, (12/78)	*P* < 0.001	46%, (91/199)
	Bilateral	35%, (42/121^a^)	85%, (66/78)	*P* < 0.001	54%, (108/199)
	Missing data	52%, (129/250)	0%, (0/78)	-	39%, (129/328)
Family History of Affected Parental Retinoblastoma	Yes	22%, (39/179^a^)	0%, (0/78)	*P* < 0.001	46%, (39/257)
	No	78%, (140/179^a^)	100%, (78/78)	*P* < 0.001	54%, (140/257)
	Missing data	28%, (71/250)	0%, (0/78)	-	22%, (71/328)
Retinoblastoma status	Affected	50%, (125/250)	100%, (78/78)	*P* < 0.001	62%, (203/328)
	Unaffected	50%, (125/250)	0%, (0/78)	*P* < 0.001	38%, (125/328)
	Missing data	0%, (0/250)	0%, (0/78)	-	0%, (0/328)
CADD score	Raw	5.7 [4.8–6.2] (250/250)	7 [5.7–8.6] (78/78)	*P* < 0.001	5.7 [4.8–6.4]
	PHRD	33 [26.6–34]	35 [33–38]	*P* < 0.001	33 [27.3–34]

^a^-excluding missing data (N-missing data)

Of the 250 individuals in the LPRB cohort, 125 (50%, CI [43.8%–56.2%]) had clinical RB and were classified as LPRB. The remaining 125 were asymptomatic carriers, giving rise to an LPRB penetrance rate of 50%.

After excluding 36 individuals under 20 years of age without offspring, and 18 with missing data on parental status, 196 *RB1* PSV carriers remained. Of these, 125 had LPRB and 71 were asymptomatic adults, yielding an age-adjusted estimated penetrance rate of 64%, CI [56.8%–70.2%].

### Parental inheritance of germline RB1 PSV among the LPRB cohort

To identify the effect of parental transmission of the *RB1* PSV on RB phenotype in offspring, a sub-cohort of the LPRB, including 145 individuals who inherited *RB1* PSVs from a carrier parent, was analyzed. After adjusting for sex of both the carrier parent and the offspring, logistic multivariate regression analysis showed that paternal inheritance of the PSV was a significant predictor for increasing the odds of developing RB (OR = 6.24, CI [3–13.8], *P* < 0.001). In contrast, the sex of the child diagnosed with LPRB was not found to significantly affect the risk of developing RB (OR for males = 1.7, CI [0.8–3.6], *P* = 0.18).

### CGRB cohorts

The three participating centers reported a total of 78 children with CGRB. Of these, 53% (41/78) were females, 85% had bilateral disease, the median age at time of diagnosis was 6.5 months [3–14] and the median raw CADD score was 7 [5.7–8.6] (Table 1).

### Clinical comparison between LPRB and CGRB cohorts

Male:female ratio was significantly higher in the LPRB cohort (males- 58% [118/204] vs females- 42% [88/204], *P* = 0.002) for all analyzed cases (symptomatic and asymptomatic carriers of *RB1* PSVs). Among symptomatic individuals in the LPRB cohort, the proportion of males was significantly higher than that of females (62% [60/97] vs. 38% [37/97], *P* = 0.002). However, male:female ratio in the CGRB cohort was almost equal (49% [43/87] vs 51% [44/87] *P* = 0.63).

Unilateral disease was significantly more frequent in the LPRB cohort compared with the CGRB cohort (65% [79/121] vs 15% [12/78], respectively; *P* < 0.001). Within the LPRB cohort, unilateral disease was also significantly more common than bilateral disease (65% [79/121] vs. 35% [42/121], respectively; *P* < 0.001). In contrast, the CGRB cohort showed the opposite trend, with bilateral disease being significantly more frequent than unilateral disease (85% [66/78] vs. 16% [12/78], *P* = < .001).

**Figure 2:**
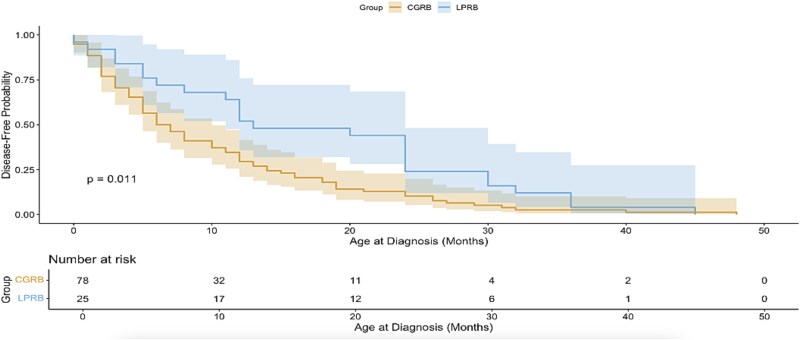
Kaplen-Meier: Time to be diagnosed with RB (LPRB vs CGRB). Blue line for the LPRB group and yellow for CGRB. Figure depicts the results of the Kaplan Meier analysis and clearly shows that LPRB cases are diagnosed at significantly older age compared with CGRB.

Kaplan–Meier analysis showed that individuals with LPRB were diagnosed at significantly older ages compared to those with CGRB, *P* = 0.01 (Fig. 2).

### *RB1* PSV locus analysis in the LPRB and CGRB cohorts

Overall, all study participants harbored 93 distinct *RB1* PSVs: 64.5% (60/93) PSVs were exclusively detected in the LPRB cohort, 33.3% (31/93) PSVs were exclusively detected in the CGRB cohort and 2.2% (2/93) PSVs were detected in both cohorts (NM_000321.3(*RB1*)c.607 + 1G > T; rs587776789; *RB1*: NM_000321.3(*RB1*):c.861G > C (p.Glu287Asp; rs1555284956). The most frequent PSV was NM_000321.3(*RB1*):c.1981C > T (p.Arg661Trp); rs137853294 detected in 18.3% of patients (60/328). Further data regarding *RB1* PSV spectrum and predicated functional characterization are shown in Table S1.

**Figure 3:**
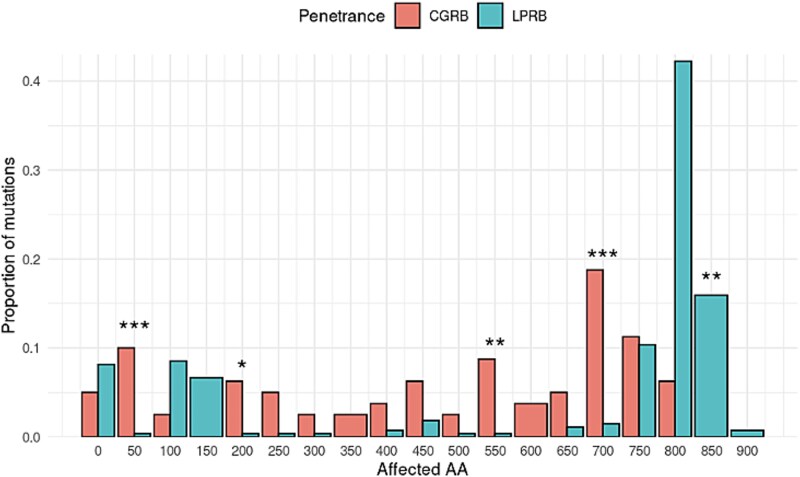
The proportion of mutations predicted to affect an amino acid, stratified by disease penetrance. The bar-plots represent the proportion of mutations in bins of 50 amino acids; bins with significantly different proportions are annotated with an asterisk. Blue for the LPRB group and red for CGRB.

A cluster of LPRB-related PSVs was noted in two gene regions: one at the 5′ end of *RB1* (amino acids 1–200) and another towards the 3′ end of *RB1* (amino acid 750 onwards). The beginning of the gene (amino acid 1–200) is functionally composed of mostly frameshift and stop-gain. LP-associated PSVs affecting the large middle stretch of *RB1* (amino acids 200–750), are notably less impactful, associating high impact PSVs in this locus with higher penetrance (Fig. 3).

### In silico functional impact of *RB1* PSVs (CADD score) comparison between LPRB and CGRB cohorts

**Figure 4:**
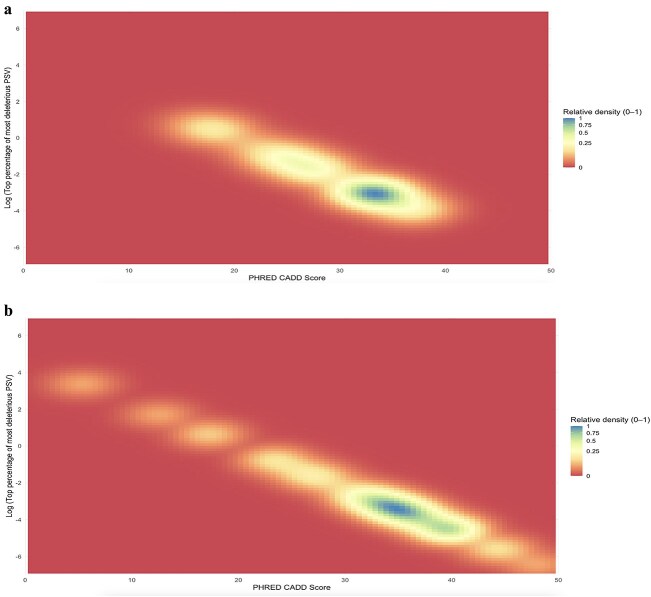
Heat map of PHRD-CADD score: (a) low penetrance retinoblastoma (b) classical germline retinoblastoma. Colors represent relative density, ranging from 0 (red) to 1 (blue).

The median raw and PHREDCADD scores, calculated across the combined LPRB and CGRB cohorts, were 5.7 [4.8–6.4] and 33 [27.3–34], respectively. While comparing the raw CADD score of each cohort, the median raw CADD score of the LPRB cohort was significantly lower than the median raw CADD score of the CGRB cohort (*P* < 0.001; Table 1). However, heatmaps of each cohort suggest a shared pathogenic threshold of PHREDCADD score of 20 (Fig. 4a and 4b).

### Multivariate logistic regression comparison of LPRB and CGRB

The following parameters were found to significantly associate with LPRB: Unilateral disease (OR = 9.3 CI [4.2–22.2], *P* < 0.001), PSVs located in the marginal regions of *RB1* Vs. non-marginal located PSVs (OR = 3.2 CI [1.4–7.65], *P* = 0.007), males (OR = 2.4, CI [1.1–5.5], *P* = 0.03) and lower raw CADD scores (OR = 1.5 CI [1.2–1.84], *P* = 0.0008).

## Discussion

In this study, our primary objective was to estimate the rate of LPRB and elucidate its underlying mechanisms by examining the clinical and molecular features of individuals carrying *RB1* PSVs. We estimated the rate of disease expression among LPRB carriers to range from 50% (non-age-adjusted) to 64% (age-adjusted), highlighting the variable expressivity of these mutant alleles. These findings suggest that LPRB may be more appropriately classified as a ‘moderate-penetrance’ condition. To the best of our knowledge, our penetrance estimate is based on the largest LPRB cohort reported to date and the most extensive collection of LPRB PSVs. Importantly, this estimate is consistent with previously reported variant-specific findings, reinforcing its validity: Hung et al. reported 36% (4/11) penetrance rate for the p.V654L missense *RB1* variant [14], and Eloy et al. observed a Disease-Eye Ratio of 0.6 for c.1981C > T/p.Arg661Trp *RB1* variant carriers [9]. Yet, for real world estimates of the penetrance of *RB1* mutant allele a prospective, multicenter study encompassing multiple ethnically and geographically diverse families is needed.

Paternal inheritance was associated with a 6.24-fold higher risk of disease expression compared with maternal inheritance, highlighting the potential epigenetic modulation of *RB1* mutant allele penetrance [9, 10, 15]. Among the known epigenetic alterations, DNA methylation is the most studied one, with specific sperm patterns stably transmitted to offspring at genetic loci encompassing *ALK* and *PHOX2B* [16–18]. In LPRB, hypomorphic *RB1* variants (e.g. p.Arg661Trp and select splice-site PSVs) have higher penetrance and tumor risk when paternally inherited due to CpG85 methylation in intron 2 that silences the paternal allele, reducing pRB to a haploinsufficient, null-like state [9, 10, 15]; maternally inherited variants retain sufficient expression to prevent tumorigenesis in most cases. Thus, systematic phasing and parent-of-origin testing are essential to refine recurrence-risk estimates and guide individualized surveillance in LPRB [19, 20].

In the current study, the LPRB cohort demonstrated a statistically significant skewed male-to-female ratio. Yet, no sex ratio differences were observed in the CGRB cohort. Furthermore, while a direct comparison of sex distribution between the two cohorts did not show a statistically significant difference, the multivariate model indicated that being male was associated with a 2.4-fold increase in the odds of developing LPRB. Large-scale epidemiological studies report inconsistent data regarding male:female ratio on RB phenotype from 0.91 (670/734) to 1.20 (2375/1976) [21–23]. This discrepancy may stem from the fact that those studies did not analyze LPRB and CGRB cohorts separately or may be attributed to regional differences in access to care rather than to biologically based sex effects [23].

A significantly higher proportion of unilateral disease was observed in the LPRB cohort compared with CGRB. These findings are supported by smaller earlier studies encompassing 34–123 cases and the GeneReviews consortium [24–26]. Combined, these suggest that LPRB is characterized by variable expressivity, and milder disease manifestations. In contrast, Salviat et al. [21] reported that LPRB is more likely to present bilaterally based on 55 patients with LPRB of a total of 1404 patients with RB. These differences highlight the need for a large, prospective multicenter study focused on LPRB, which could help clarify the clinical features unique to LPRB.

Another clinical aspect that differs between LPRB and CGRB is age at diagnosis: LPRB is associated with older presentation age. These findings were supported by others and further reinforced the classification of LPRB as milder, delayed onset, disease [9, 21]. Interestingly, the clinical presentation of LPRB resembles that of sporadic RB, where affected individuals typically present at an older age with unilateral disease. This similarity has important implications for clinical management, particularly in terms of surveillance strategies and timing of diagnosis.

The effect of PSV locus in the *RB1* gene on disease penetrance shown herein- a clustering of PSVs located to two distinct regions is worth mentioning: PSV affecting these protein regions were more commonly associated with LPRB. Such clustering may partially be explained by rescue of the mutant transcribed allele from nonsense-mediated decay (NMD), a common determinant of incomplete penetrance in many diseases [27]. Moreover, a PSV-location-specific-phenotype has been shown in other cancer susceptibility genes such *APC* and *BRCA1 BRCA2*, specifically in gene loci that are rescued from NMD and thus maintain a partial protein activity. In support of that observation, PSVs in exons 24 and 25 of the *RB1* gene are known to produce low-penetrant disease, even when completely deleted [28, 29].

To the best of our knowledge, this is the first study to apply in silico pathogenic predication score analyses on LPRB PSVs. This analysis revealed that these PSVs not only have significantly lower CADD scores than CGRB but also cluster more frequently in the N- and C-terminal regions of pRB, outside the canonical ‘pocket’ domain. Together, these findings support a hypomorphic model of partial function loss. Furthermore, we propose a preliminary CADD threshold of 20 as a potential benchmark for future genotype–phenotype stratification, recognizing that this requires validation in independent cohorts.

This study introduces the first multivariate model that simultaneously accounts for clinical presentation and molecular features in LPRB. The model identified male sex, unilateral presentation, lower CADD scores, and specific PSV topography as independent features of LPRB, underscoring the novel contribution of raw CADD scores and protein-domain mapping to clinical risk stratification. Building on these findings, we propose a combined framework that incorporates domain location, parent-of-origin effects, and NMD-escape predictions to refine penetrance estimates and guide individualized surveillance strategies. These associations, however, require validation in functional assays (e.g. pRB–E2F binding) and confirmation across diverse, prospective cohorts before clinical implementation.

The study has several limitations. First, by including only individuals with sequencing results, we excluded obligate carriers and clinically diagnosed cases. This introduces selection bias and limits the generalizability of our findings to the broader LPRB population. Second, although our CGRB cohort includes children with confirmed germline *RB1* PSVs, parental sequencing was not universally carried out. Consequently, undetected LPRB cases may have been included in that cohort. Given that LPRB is rare, and represents approximately 5–10% of germline *RB1* PSV carriers [3, 26], the likelihood of such inclusion is low. To further mitigate that possibility, we excluded RB cases with a family history of RB. Notably, the clinical features of our CGRB cohort (bilaterality, early age at diagnosis, absence of sex predilection) closely align with those of a large, independently characterized CGRB cohort [21], providing further indirect support for its representativeness of CGRB while acknowledging residual uncertainty. Third, a portion of our data was derived from previously published clinical sources, which often contained missing information (e.g. sex, family history of RB and laterality). Our use of complete case analysis may have introduced bias and reduced statistical precision. Fourth, the retrospective, multi-institutional nature of the study, combined with variability in diagnostic criteria and screening protocols, may have exposed our genotype–phenotype correlations to unmeasured confounding factors. Finally, although we propose that the observed paternal bias may be related to methylation at the intron 2 CpG85 island, we did not perform direct molecular assays on patient samples.

In summary, we estimated the penetrance of LPRB to range from 50% (non-age-adjusted) to 64% (age-adjusted), reflecting moderate expressivity of *RB1* PSVs while showing a 6.3-fold higher risk for developing RB when the mutant allele is paternally inherited. Clinically, LPRB is hallmarked by predominantly unilateral tumors, a high male:female ratio and older age at diagnosis compared with CGRB. At the molecular level, hypomorphic *RB1* variants in LPRB tended to cluster in the N- and C-terminal regions of pRB and exhibited lower in silico pathogenicity scores, consistent with partial rather than complete loss of RB protein function. Together, these findings define a distinct clinical and molecular profile for LPRB, with significant implications for genetic counseling, risk stratification, and tailored surveillance. In light of these insights, we propose a shift in terminology from ‘low penetrance RB’ to ‘moderate penetrance RB,’ to better reflect its clinical and biological behavior.

## Materials and methods

### Study definitions

Asymptomatic carrier: An individual with a pathogenic *RB1* PSV but no clinical signs of RB. LPRB: A patient with clinical RB within a family where other *RB1* PSV carriers remain unaffected. CGRB: A patient with germline *RB1* PSV in the context of full or typical disease penetrance.

### LPRB cohort

To assemble the LPRB cohort, a systematic literature search using both PubMed (https://pubmed.ncbi.nlm.nih.gov) and Google Scholar (https://scholar.google.com/) was carried out including papers published from 1997–2024. Search terms included ‘low penetrance *RB1* mutations’, ‘incomplete penetrance *RB1* mutations’, and ‘phenotypic variability of *RB1* alleles’. Studies were eligible for inclusion if they met the following criteria: (1) reported families in which at least one individual with RB and one or more asymptomatic carriers harbored the same germline *RB1* PSV; and (2) provided molecular confirmation of the variant by DNA or RNA sequencing. Studies were excluded if they lacked genetic confirmation, did not report disease status of variant carriers, included only somatic mutations or included more than a single PSV per individual (Fig. 1). For each reported individual, demographic characteristics (i.e. sex, age at diagnosis, affected parent’s sex) and clinical data (i.e. disease status, laterality- unilateral or bilateral) were extracted.

### CGRB cohort

This cohort included children who were diagnosed with RB between 2019–2024. Data was obtained from three medical centers: Sheba Medical Center (Ramat Gan, Israel), Seoul National University College of Medicine (Seoul, Republic of Korea), and Ramathibodi Hospital, Mahidol University (Bangkok, Thailand). Children were eligible for inclusion if a germline testing of peripheral blood leukocytes, performed by DNA or RNA sequencing, confirmed a PSV in the *RB1* gene. Additionally, we excluded (1) RB cases with a family history of RB and (2) individuals harboring more than two distinct PSVs in *RB1*, to avoid confounding from complex genotypes.

### Penetrance rate of LPRB

The following formula was used to estimate the rate of LPRB: 


\begin{equation*} {Penetrance\ rate\ of\ LPRB} =\frac{\sum LPRB}{\sum LPRB+\sum Asymptomatic\ carriers.} \end{equation*}

First, we applied a non-age-adjusted estimation, including all individuals within the LPRB cohort. In a second, age-adjusted analysis, we excluded asymptomatic individuals who were either under 20 years of age or had no children, to account for the possibility that some might still develop the disease. This approach ensured that all included participants had reached, or surpassed, reproductive age. To ensure reproductive age was reached, individuals with missing information regarding offspring were also excluded, particularly in studies that did not provide detailed pedigree data. This approach aimed to reduce the risk of misclassifying individuals who had not yet completed the risk period for developing RB. The penetrance rate was then recalculated using the same formula, adjusted to age and included only asymptomatic individuals and asymptomatic adult carriers.

### Parental inheritance of germline *RB1* PSV

To assess the risk of RB development among low-penetrance carriers (individuals with an inherited *RB1* PSV from a parent with the same variant), we performed a multivariate logistic regression analysis, adjusting for patient and parental sex.

### *RB1* PSVs loci comparison analysis

Over-representation of PSVs was assessed using chi-squared testing, comparing the proportions of low-penetrance variants or paternally inherited alleles across predefined loci within the gene.

### In silico functional impact of *RB1* PSVs comparison

**Figure 5:**
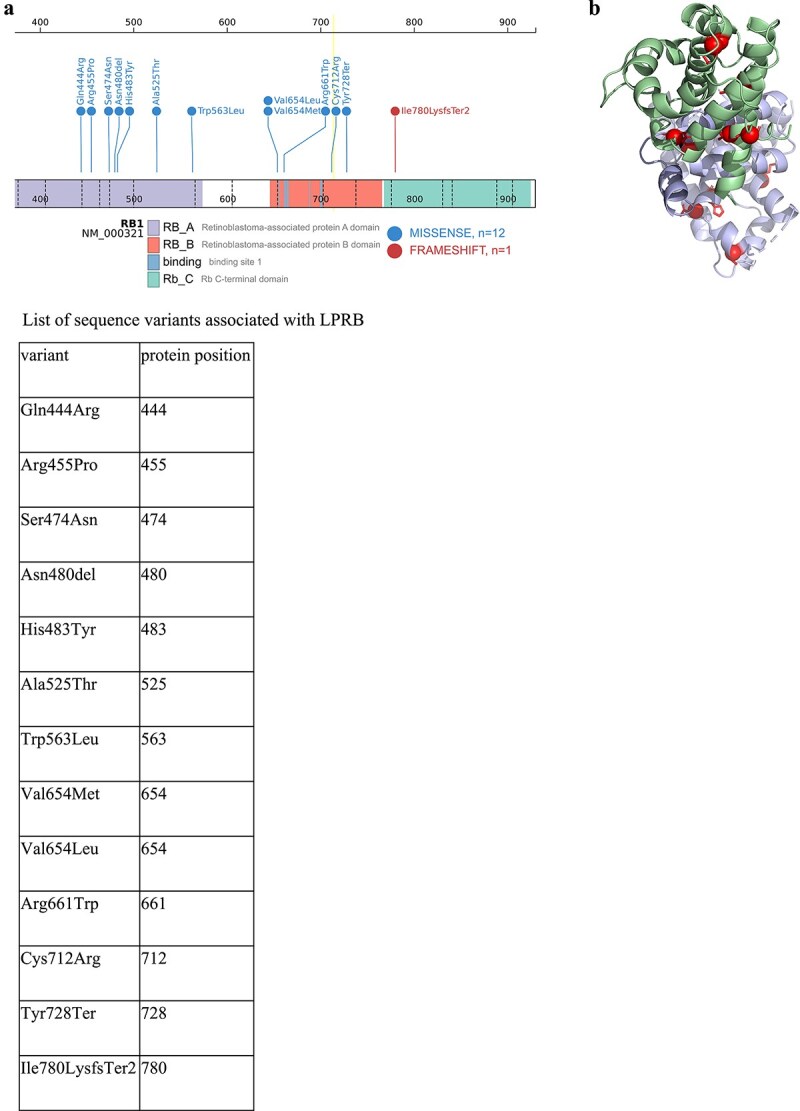
(a) A schematic representation of the location of sequence variants along the pRB associated with LPRB. (b) the RB1 protein binding pocket (residues 380–577 purple, 645–785 green), with low penetrance mutations marked by red orbs. List of sequence variants associated with LPRB.

The pathogenic potential of each PSV was predicted in silico using the Combined Annotation Dependent Depletion (CADD) score (version 1.7), which predicts the deleteriousness of single nucleotide variants, multi-nucleotide substitutions, as well as insertion/deletions variants in the human genome, including splicing regions [11]. While the raw CADD score was used for analyses, a logarithmic transformation—the PHREDCADD score, was applied for setting a pathogenicity cutoff [11]. In this study, we applied a PHREDCADD score cutoff of > 20, consistent with the cutoff recommended by the algorithm’s developers [11] and supported by the distribution of values in our dataset. For single nucleotide variants, we used Ensembl’s Variant Effect Predictor (VEP)—a comprehensive genome annotation database and analysis platform that provides a wide range of bioinformatics tools for genomic data analysis, including the CADD algorithm score [12]. We relied on Ensembl to obtain genomic coordinates, relevant transcripts, and predicted functional consequences for the PSVs analyzed. For more complex PSVs we directly used the CADD portal (https://cadd.gs.washington.edu/). Simplified protein mutations visualization was created using proteinpaint (https://viz.stjude.cloud/tools/proteinpaint) (Fig. 5); A functional domain-annotated mutation location was generated by PyMol (https://www.pymol.org/) (Fig. 5).

### Multivariate logistic regression comparison of LPRB with CGRB

To identify independent predictors of penetrance status (LPRB vs. CGRB), we performed a multivariate logistic regression, including disease laterality (unilateral/bilateral), patient sex, raw CADD score, and PSV location along the RB protein (marginal: amino acid 1–200 or > 750 Vs. non-marginal).

### Ethics

The data were collected in accordance with the relevant local ethics committees and their regulatory frameworks. Additionally, all data in this study were de-identified according to the Health Insurance Portability and Accountability Act safe-harbor privacy rules [13], with no risk to individual privacy. The requirement for informed consent was waived by the local ethics committees.

### Statistical analysis

All statistical analyses were performed using RStudio (R version 4.2.2). Categorical variables were represented by percentage while continuous variables were represented by mean and standard deviation (SD) values if distributed normally, and otherwise by median and interquartile range (IQR) values. We set the significance level to 5% (two-tailed) and the confidence interval (CI) to 95%. For multivariable logistic regressions, we used the generalized linear logistic model to estimate odds ratios (ORs) and 95% CIs. We compared the age at RB diagnosis between LPRB and CGRB groups using Kaplan–Meier survival analysis, The log-rank test was used to assess differences between the groups.

## Supplementary Material

TableS1_ddag026

## Data Availability

The datasets generated and/or analyzed during the current study are available from the corresponding author on reasonable request.

## Web resources

PubMed- https://pubmed.ncbi.nlm.nih.gov

Google Scholar- https://scholar.google.com

Ensembl Variant Effect Predictor- https://www.ensembl.org/Tools/VEP

Combined Annotation Dependent Depletion portal- https://cadd.gs.washington.edu/
